# Cocaine Induced Vasculitis: Have We Found a Culprit?

**DOI:** 10.1155/2012/982361

**Published:** 2012-12-30

**Authors:** Alfredo Sánchez-Cruz, Sylmarie Marrero, José Betancourt, Myrna Andino, Adolfo Lopez, Jose Gutierrez-Nuñez

**Affiliations:** ^1^Department of Internal Medicine, San Juan City Hospital, San Juan, PR 00928, USA; ^2^Department of Internal Medicine, VA Caribbean Healthcare System, San Juan, PR 00921, USA

## Abstract

Cocaine abuse is relatively common in our society. To enhance profitability and acceptability of the product, it is not uncommon for illicit drugs to undergo several processes. The Drug Enforcement Agency (DEA) has reported that seventy percent (70%) of cocaine seized at USA borders has been adulterated with levamisole, previously used as chemotherapeutic and immunomodulator for several conditions. Among the side effects of levamisole-adulterated cocaine, necrotizing vasculitis is the more dramatic. We report three cases of necrotizing vasculitis associated with antineutrophils cytoplasmic antibodies (ANCAs) positivity, linked to the use of cocaine. To our knowledge, these are the first cases of cocaine induced vasculitis reported in the Caribbean.

## 1. Introduction

Cocaine abuse is relatively common in our society. Recently, the Drug Enforcement Agency (DEA) has reported that seventy percent (70%) of cocaine seized at United States (US) borders has been adulterated with levamisole [1]. This agent, which is an antihelminthic and chemotherapeutic drug, indirectly increases the number of D1 dopamine receptors in the brain and has a cholinergic effect that seems to potentiate the effect of cocaine. It also has immune stimulating effect producing antineutrophils cytoplasmic antibodies (ANCAs). Several side effects have been associated with its use, including severe neutropenia and necrotizing vasculitis. Levamisole is a challenging drug to test; it has a half-life about 5.6 hours and specific testing is not routinely available [2]. Recently, several cases with this manifestation have presented in our institution. Although cases have been previously reported in the USA, none had been reported in Puerto Rico or the Caribbean.

## 2. Case Report

 A 45-year-old male cocaine user without any previous systemic illness complains of progressive painful erythematous lesions in both auricles and extremities, constant burning, 10/10 pain in upper and lower extremities, although more prominent in upper extremities. Patient also refers suffering of unquantified fever, congested nose, and fatigue; denies nausea, vomiting, and shortness of breath or changes in mental status. Four days prior to admission, patient was evaluated in a health clinic for the above-mentioned lesions and was referred for a follow-up visit to surgery for a skin biopsy. One day prior to admission, patient was taken to the same clinic for the worsening of his skin lesions; he was transferred to our institution for further evaluation and management.

**Figure 1:**
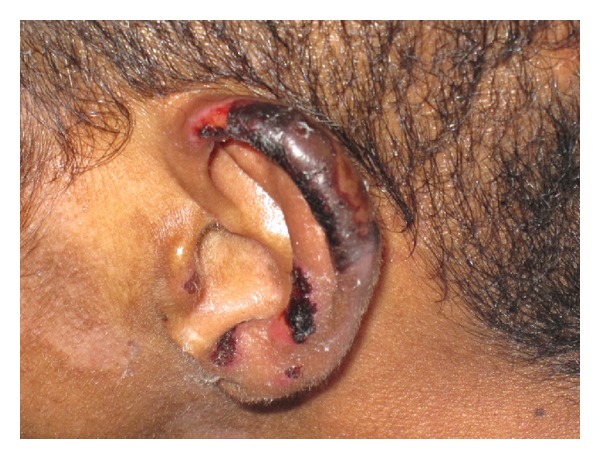
Magnified view of purpuric retiform lesion.

History was remarkable for smoking (use of) cocaine; last reported use was 10 days prior to evaluation. Physical examination demonstrated several purpuric retiform patches with erythematous borders in the helix of both ears and both upper and lower extremities (Figure 1). A working diagnosis of vasculitis was made with suspicion of cryoglobulinemia; high-dose steroids were initiated. Toxicology test in urine was negative for cocaine; hepatitis profile and HIV test were nonreactive.

**Figure 2:**
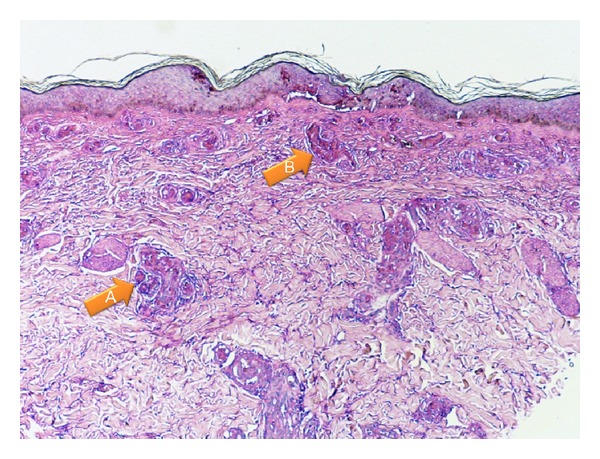
A.intravascular thrombosis B. Perivascular lymphocytic infiltrates.

**Figure 3:**
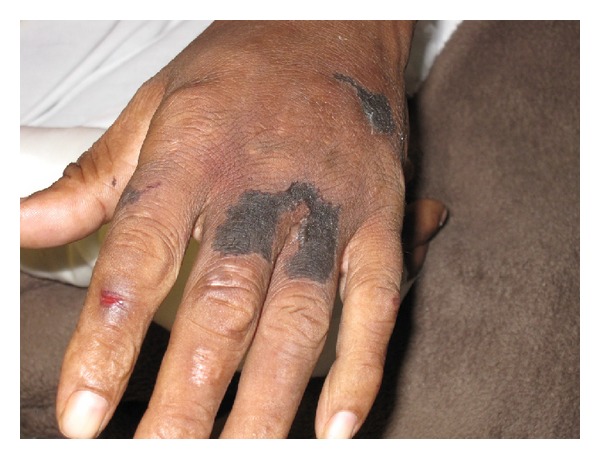
Almost clinical resolution after 30 days of being cocaine-free.

C-ANCA (1.7 IU/mL), P-ANCA (1.7 IU/mL), ANA, and cryoglobulins were positive. Complements levels were decreased C3 = 92.50 mg/dL and C4 = 12.30 mg/dL. WBC = 4.7 × 10^3^/uL, Hb = 10.2 g/dL, Hct = 29.8%, and platelets = 390 × 10^3^ uL. Skin biopsy (punch) demonstrated intravascular thrombosis and mild perivascular lymphocytic infiltrates (Figure 2). High-dose steroids were continued and plasmapheresis was initiated. Complete clinical resolution of skin lesions was noted after treatment and thirty (30) days of no cocaine use (Figure 3). 

**Figure 4:**
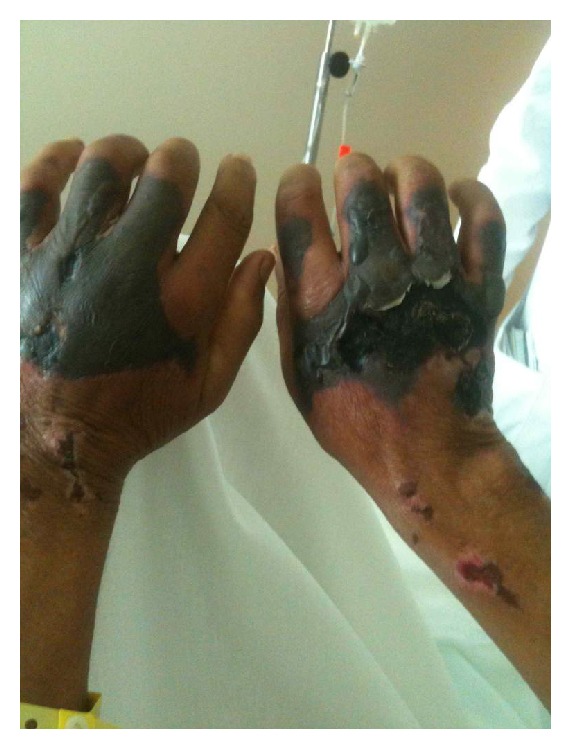
Necrotic lesions with erythematous base.

The second patient is a 52-year-old male with medical history of constant crack cocaine and marihuana abuse, complaining of bilateral upper and lower extremities necrotic skin lesions, which were also found on ears, nose, and genital area associated with a burning, 10/10 pain of two (2) weeks of evolution. Chills, nauseas, and vomits were referred as associated symptoms; patient denied fever, shortness of breath, or changes in mental status. History remarkable for smoking cocaine, last reported use was the night before initial evaluation at the emergency room. Physical examination showed necrotic lesions with an erythematous base, tender to palpation, on both ears, nose, upper and lower extremities, and genital areas (Figure 4). Laboratory evaluation was remarkable for neutropenia: WBC = 3.5 × 10^3^/uL, microcytic anemia, Hb = 9.92 g/dL, Hct = 28%, and platelet count of 290 × 10^3^ uL. Due to the appearance of necrotic tissue, a diagnosis of vasculitis with infected necrotic ulcers was made. High-dose steroids and broad-spectrum antibiotics were initiated. Toxicology test was positive for cocaine and cannabinoids, while the hepatitis profile and HIV test were nonreactive. C-ANCA (2.55 IU/ml), P-ANCA (1.69 IU/ML), and cryoglobulins were positive. ANA was negative in this occasion. Complements levels were decreased, C3 = 55.30 mg/dL, and C4 = 8.86 mg/dL. As with the previous patient, skin biopsy revealed homogenous eosinophilic material with blood vessels and lymphocytic infiltrate in dermis; however, no evidence of intravascular thrombosis was found. As recommended by hematology service, plasmapheresis was started, and antibiotics and high-dose steroids were continued. Clinical resolution was observed, but patient left against medical advice before the treatment was completed.

**Figure 5:**
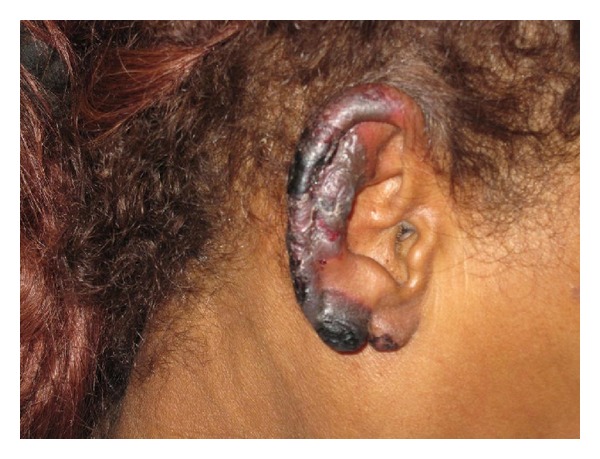
Purpuric retiform patches with erythematous borders and necrotic tissue.

The third patient is a 60-year-old homeless female who complained of suffering 8/10, burning pain in both ears for about six months prior to the initial evaluation, and that exacerbated since the day before arrival to our institution. Patient denied any associated symptoms. History of smoking (use of) cocaine: last reported use was the day of admission. Physical examination remarkable for purpuric retiform patches with erythematous borders and necrotic tissue on both ears (Figure 5). Purpuric retiform patches also observed on the tip of the patient's nose and inferior aspect of second to fifth right toe. Patient also presented with foul smelling necrotic ulcer in the distal right, lower extremity. Patient was admitted with broad-spectrum antibiotics for her ulcer but high-dose steroids were not initiated. Laboratory data showed neutropenia, WBC = 3.6 × 10^3^/uL, and microcytic anemia, Hb = 11.3 g/dL, Hct = 34.2%, and Plts = 368 × 10^3^ uL. Toxicology was positive for cocaine: yet, hepatitis profile and HIV test were nonreactive. Complements levels C3 (131 mg/dL) and C4 (24 mg/dL) were within normal values. A skin biopsy revealed vascular thrombosis in the small-sized vascular channels in the dermis. P-ANCA and C-ANCA levels could not be obtained. Although clinical resolution was observed during hospitalization, patient decided to left against medical advice.

## 3. Discussion

Cocaine abuse affects more than five (5) million people in the USA with side effects that are not limited to the parent drug. To enhance profitability and acceptability of the product, it is not uncommon for illicit drugs to undergo several processes. Among these, adulteration (intentional addition of a substance with similar pharmacologic properties which attenuates the effects of the parent drug) [4] presents as a new challenge to the medical community. 

Levamisole is an anthelmintic agent used in veterinary medicine that was formerly used as an adjuvant with fluorouracil in the treatment of colorectal cancer; as immunomodulator in rheumatoid arthritis; as treatment in nephrotic syndrome. It is known for augmenting dopamine and endogenous opiates levels in the brain, increasing D1 dopamine receptors, and producing cholinergic effects [5]. Levamisole may inhibit MAO and catechol-O-methyltransferase activity, prolonging the presence of catecholamine neurotransmitters in the synapse, adding to the reuptake-inhibition effect of cocaine, and augmenting its effects (some cases of mood elevation have been reported in humans as a side effect of adjuvant therapy with levamisole for colon carcinoma) [6].

Levamisole's immune stimulating effect leads to the production of autoantibodies (antinuclear and antineutrophils antibodies), which leads to ANCA positivity (presented in the first two patients), severe neutropenia, and focal necrotizing features of vascular damage [3]. Levamisole-induced, occlusive, necrotizing vasculitis is an uncommon side effect of this agent [4]. It presents as purpuric retiform lesions with necrotic patches typically manifesting on the ears and extremities, as previously reported in patients who received treatment with levamisole for nephrotic syndrome. Also, it differs from lesions directly related to pure cocaine described as palpable purpura or Wegener's granulomatosis like [7]. Microscopically, levamisole can produce a mixed pattern of leukocytoclastic vasculitis, thrombotic vasculitis, and/or vascular occlusion as seen in skin biopsies from children using levamisole for the treatment of nephrotic syndrome [8].

Although levamisole levels were not measured because of its short half-life of 5.6 hours and because routine tests for its detection are not commonly available, we believe that the adulteration of cocaine with levamisole was the cause of the clinical presentation of the three evaluated patients. 

Retiform purpuric lesions that later become necrotic, an histopathologic report of intravascular thrombosis with a mild perivascular lymphocytic infiltrates, and ANCA positivity in a patient with recent history of smoking (use of) cocaine should raise the suspicion of a levamisole-induced vasculitis. Treatment is primarily supportive, however steroids have been used in some cases with success [4]. Since their initial evaluation and treatment, the first two patients have been admitted in several occasions for the same lesions; the use (smoking) of cocaine was reported prior to every admission, thus linking cocaine use to the appearance of these lesions. Clinicians should recognize this presentation and be aware that the discontinuation of cocaine use, more than the use of immunosuppresors, leads to the resolution of these lesions.
